# The effects of inflow of agricultural biogas digestate on bivalves’ behavior

**DOI:** 10.1007/s11356-021-15199-1

**Published:** 2021-07-12

**Authors:** Jasper Tembeck Mbah, Joanna Chmist-Sikorska, Krzysztof Szoszkiewicz, Wojciech Czekała

**Affiliations:** 1 Department of Ecology and Environmental Protection, Poznań University of Life Sciences, Wojska Polskiego 28, 60-637 Poznań, Poland; 2 Polish Institute of Meteorology and Water Management - National Research Institute, Warsaw, Poland; 3 Department of Biosystems Engineering, Poznań University of Life Sciences, Poznań, Poland

**Keywords:** Water monitoring, Water pollution, Bivalves, Bioindication, Digestate, Early warning systems, Valvometry

## Abstract

This study focused on the reaction of bivalve molluscs to biogas digestate, which is a waste product of an increasingly developing biogas production in rural areas worldwide. The effects of biogas digestate on aquatic organisms are not fully known, and neither this substance nor any types of manure were tested in the monitoring based on valvometry, which is a biomonitoring method based on bivalve behavior. The change in bivalves functioning in biogas digestate inflow was studied using three different diluted digestate concentrations. Exposure to the highest concentration of digestate induced a decline of mean shell opening and activity time of *Unio tumidus* species. A significant difference in behavioral patterns was recorded during the first 10 min after exposure to the digestate. A Gradual decreasing tendency of shell opening levels was apparent under the highest concentration reaching 55% compared to the pretreatment value. Also, a decreasing tendency was observed under the medium concentration (82.4% of initial level) after 2 h, while an increase in shell opening levels was recorded in the most diluted digestate. This research work proved that the inflow of biogas digestate has significant impact on bivalves’ behavior. *Unio tumidus* is a sensitive indicator of biogas digestate inflow in the aquatic environment. Moreover, it proved that the opening and closing activities over time depend on the concentration of the digestate. Therefore, the mollusk bivalves might be utilized in early warning systems to detect organic pollutants in water.

## Introduction

In recent years, bioindication and biomonitoring have become promising methods for studying the impacts of environmental factors on ecosystems (Parmar et al. 2016). Over the years, our knowledge of bioindicators has increased to assist in studying various types of environments using different taxonomic groups. Biomonitoring techniques based on living organisms that respond to environmental changes with detectable and reproducible ways are already used in practice to test freshwater, marine, and wastewaters (Parmar et al. 2016; Schöne and Krause Jr 2016; Zhou et al. 2008).

The efficient monitoring of water resources is regarded as fundamental for effective management of water quality and aquatic ecosystems because, aquatic ecosystems are subject to several anthropogenic disturbances, including various environmental toxicants (Parmar et al. 2016). Continuous water quality monitoring basing on the reaction of bivalves can determine water quality considering various physicochemical factors, contaminants, or toxic substances (Bae and Park 2014; Kramer and Foekema 2001a, b; Salánki et al. 2003). They play a particularly important role in controlling the quality of drinking water for municipal water supply due to their great impact on the health of consumers. For this reason, several Biological Early Warning Systems (BEWS) have been developed based on the response behavior of organisms to continuously detect a range of pollutants for effective water quality monitoring and management (Bae and Park 2014; Chmist et al. 2018).

Many freshwater organisms have been used in biological monitoring including mussels, macroinvertebrates, fish, bacteria, algae, and vascular plants (Bae and Park 2014; Chmist et al. 2019; Błachuta et al. 2014; Brabec and Szoszkiewicz 2006; Mazur et al. 2016, 2018). Benthic macroinvertebrates show sensitivity to many physical, chemical, and biological disturbances occurring in the aquatic ecosystems such as heavy metals, hydro-morphological degradation, nutrient enrichment, and acidification (Błachuta et al. 2014; Li et al. 2010; Mazur et al. 2016, 2018). These organisms are excellent passive biomonitors, accumulating both organic and inorganic substances (Salánki et al. 2004; Vinas et al. 2012), and active monitoring using a behavioral reaction to pollution (Bae and Park 2014; Salánki et al. 2004). Despite the large potential of mussels in environmental monitoring, this group of organisms is underrepresented in standard ecotoxicological methods (Hartmann et al. 2016).

Bivalve molluscs have been explored in ecotoxicology for more than 20 years (Hartmann et al. 2016; Metcalfe and Charlton 1990), and the technique using their behavioral reaction (opening and closing activities over time) for the estimation of water quality is called valvometry (Sow et al. 2011). Mussels react differently to various substances, and they are not equally sensitive to all stressing factors. Using the recognized shell opening and closing pattern of mussels, it is possible to monitor water quality. With contaminant in water, mussel will close their shell either totally or partially depending on the concentration of the contaminant, while in the absence of contaminants, mussel keeps their shells more open (Bae and Park 2014; Chmist et al. 2019). As filter feeders, mussels are particularly susceptible to water, organic, and inorganic chemicals (Hartmann et al. 2016).

Biogas digestate is a type of fertilizer used in agriculture, and the volume of its production has significantly increased in recent years since biogas production has become an important direction for the development of renewable energy source and sustainable development of rural areas (Czekała et al. 2020; Sogn et al. 2018). Biogas digestate is a fertilizer produced next to biogas as a result of anaerobic digestion (Czekała 2019). This liquid fertilizer is characterized by high nutrients content (Czekała et al. 2020). Their content and types are closely related to the kinds of substrates used in biogas production. In the case of irrational management, digestate can be a threat to the environment. Agricultural biogas plants produce biogas and digested pulp which is mostly used as a liquid fertilizer for agricultural soils although it can be converted to solid fertilizer (Czekała et al. 2017). The digestate is most often used for fertilizing. However, attention should be paid to possible threats in the ecosystems resulting from its application (Nag et al. 2020).

This study focused on the effects of biogas digestate from agricultural biogas plants on aquatic organisms. Due to the development of biogas production in rural areas, this organic substance will be produced in increasing quantities, and environmentally safe methods of its use must be developed. Biogas digestate effects on aquatic organisms are still not clear, and neither this substance nor any type of manure or other organic waste was tested in the monitoring based on valvometry so far. The aim of this study was to evaluate the effects of biogas digestate on *Unio tumidus*, to reveal the potential of bivalve molluscs in monitoring increasingly dangerous elements of agricultural wastewater. The reactive time of bivalves with regards to the inflow of different concentrations of biogas digestate was checked, with a focus on the short-term reaction to test its ability to deliver signals for the early warning systems. It was hypothesized that (1) the inflow of biogas digestate has a significant effect on bivalves’ behavior and (2) the behavior of bivalves depends on the concentration of biogas digestate content.

## Methods

### Monitoring system

This study based on laboratory experiments was conducted on the Biological Early Warning System known as SYMBIO, which utilizes mussels as bioindicators (Chmist and Szoszkiewicz 2017; Chmist et al. 2019). The system facilitates precise observations of changes in mussel behavior, using an 8-mm magnet placed on one shell-half while the other shell-half is attached to a methacrylate holder using adhesive. Furnishing mussels with magnets does not influence measurements because the shells are fitted with magnets, during the clean water monitoring phase (control period) and after adding pollution (treatment period).

Mussels were treated in a 150 × 25 × 25 cm glass aquarium (tank) filled with 60 L of bottled water. A controller with eight sensor-computer connections (props) connects each of the eight bivalves to the computer, and each experiment used eight mussels. To guarantee aeration and ensure optimal oxygen condition, extensive tubes connected to an air pump were used to provide oxygen at different locations within the aquarium. An aquarium chiller is used to keep the water temperature constant at 14.7°C ± 0.2°C. The pH of the water ranged between 7.6 and 8.4, and dissolved oxygen (DO) between 7.8 and 8.1 mg‧L^−1^. No mortality was observed during the acclimatization period. The experiment used the native bivalve species swollen river mussel (*Unio tumidus*) as the indicator organism. Mussels were collected before the experiment from a lake in Wielkopolska province, Poland. The selected aquatic ecosystem was in a good ecological status so that the biological material for monitoring would not be subjected to significant water degradation. All bivalves used were estimated to be adults between the ages 6 and 9 years and uniform in size (N = 40, 6±1 cm length and 3.5 ± 0.5 cm width) (Jakubik and Lewandowski 2016).

Individual specimens were fixed in the aquarium on methacrylate holders and attached to sensors. The Hall sensor measured changes in magnetic field strength. The shell openings and closure influenced the distance between the magnet and the sensor, thus changing magnetic field strength. The entire process was recorded by the sensor that processes data. Additionally, a specially designed monitoring software facilitated visualization and recording of all behavioral events taking place during the system operation. A computer with dedicated software is connected to every sensor and collects continuous data of shell opening level (fifty times a minute). Approximately 72,000 records of shell positions were identified every day.

### Biogas digestate

Biogas digested pulp was obtained from one of the largest biogas plants located in Poland. Maize silage and cow slurry were the substrates used to produce biogas. The pH of biogas digestate was 8.17, the nitrogen content was 3.73 kg·Mg^-1^ (2.56 kg·Mg^-1^ NH3-N), and phosphorus content (P_2_O_5_) was 1.06 kg·Mg^-1^. The results were determined in the fresh matter. The contents of heavy metals and microbiological contaminants in the digestate were analyzed in accordance with the national regulation (Regulation of Ministry of Agriculture and Rural Development 2008). The allowed values of heavy metals and microbiological contaminants were not exceeded in the digestate used for this research.

### Experiment design

Three separate experiments were completed using different concentrations of biogas digestate applied. The biogas digestate was diluted with water in the following proportions: 1:400, 1:200, and 1:100.

Before the digestate treatment, bivalves were kept in clean water with temperatures ranging between 10 to 12 ± 1°C for a 7-day acclimation, 1 h before the treatment period is considered the control phase. The mean shell opening levels during this hour are taken as a reference to the treatment period, and the measured results are standardized to this level (the control period is considered 100%). During both the acclimation and exposure periods, the bivalves were not fed but kept in water saturated with dissolved oxygen.

Bivalves were exposed to biogas digestate for 10 h, and behavioral observations recorded 50 times per minute. The mean shell opening of eight mussels used in each experiment was plotted on a graph. In this way, it was possible to observe changes in the shell opening level during the control and treatment periods under different concentrations of biogas digestate.

Behavioral differences between different concentrations of biogas digestate were also analyzed, at various time intervals. During the first hour, the shell opening level changes were analyzed in 10-min intervals and 1-h intervals for the rest of the experiment. The mean value differences for every studied time interval was verified with ANOVA analysis using the post hoc test LSD (least significant differences).

The chemical content of the water was analyzed. Water samples were collected during the treatment period (after biogas digestate application and at the end of the experiment), and total phosphorous and nitrogen were analyzed for each sample.

## Results

### General tendencies

Biogas digestate was applied in three different concentrations (1:400, 1:200, and 1:100 dilution), and the changes in their chemical contents were analyzed. The total form of phosphorous and nitrogen is presented in Table 1. The reduction of total phosphorus and nitrogen in water after biogas digestate application was different for each of the concentrations. The highest phosphorus reduction was observed in the dilution 1:200 and in 1:100 in the case of total nitrogen. However, the deduction of nitrogen and phosphorus was smallest at dilutions 1: 200 and 1:400 respectively.

**Table 1:** Content of total phosphorous (P) and total nitrogen (N) after the digestate application and after 14 days (concentrations in mg/L)

Manure concentration	Total P		Total N	
	After application	End of the experiment	After application	End of the experiment
1:400	2.61	2.29	7.55	6.44
1:200	4.55	3.88	8.10	7.25
1:100	7.67	7.28	9.54	8.49

**Fig. 1:**
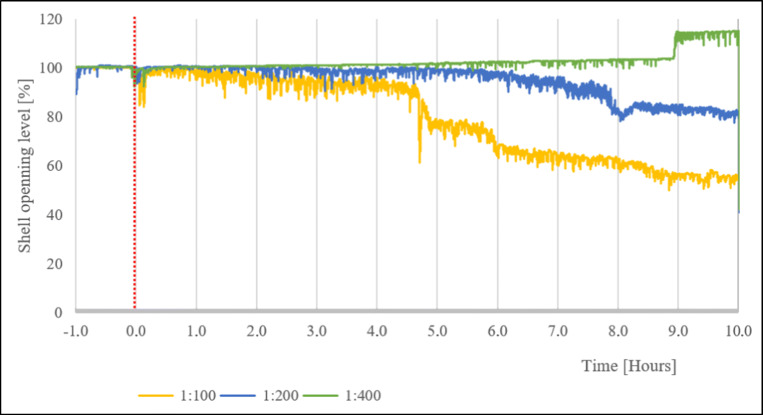
Mussel reaction to digestate application in the concentration 1:400, 1:200, and 1:100. Red line—time of manure application

The general tendency for the mussel reaction to biogas digestate during the 10 h is shown graphically in Fig. 1. The curves represent the mean shell opening of eight mussels used in each of the three different biogas digestate dilutions: 1:400, 1:200, and 1:100.

Shell opening level decreased during the first few minutes after each biogas digestate application. The decreasing tendency of the shell opening was apparent under the 1:100 dilution reaching 55% compared to the reference value. A decreasing tendency was also recorded under the dilution 1:200. The most diluted biogas digestate (1:400) did not reduce the shell opening level of bivalves (except for initial short-term event) and, an increasing tendency was observed, especially during the 9th hour after digestate application.

### The early warning reaction during the first hour

During the first hour, shell opening changes were analyzed in 10-min intervals. Mean values and variability (standard deviation) are presented in Table 2. ANOVA analysis confirmed that differences between mean values of various time intervals are highly significant (p<0.01) for each digestate concentration (Table 3). ANOVA analysis also confirmed that differences between mean values of various time intervals are highly significant (p<0.01) for each biogas digestate concentration (Table 3). The LSD tests showed that differences between most means of 10-min intervals appeared significant (p<0.05). The non-significant differences were limited for dilution 1:400 between 40 and 60 min, 1:200 between 30 and 40 min, and between 30 and 60 for 1:100.

**Table 2:** The shell opening level changes during the 1-h period after digestate application. Explanation: *mean***,** the mean shell opening level (mean); *SD*, standard deviation; *n*, number of shell opening measurements recorded; digestate dilutions: 1:100, 1:200, and 1:400

Time interval (min)	n	1:100		1:200		1:400	
		mean	SD	mean	SD	mean	SD
Before application	3189	100.00	0.07	100.00	1.66	100.00	1.05
0–10 min	532	93.94	4.49	98.60	1.67	98.40	1.84
10–20 min	532	98.22	0.47	99.98	0.31	99.28	0.42
20–30 min	532	99.16	0.49	100.17	0.17	99.90	0.18
30–40 min	531	99.17	0.64	100.29	0.21	100.19	0.09
40–50 min	532	99.03	0.81	100.54	0.16	100.21	0.09
50–60 min	532	99.18	1.02	100.33	0.25	100.27	0.05
Total	6380	99.06	2.14	99.99	1.36	99.85	1.05

**Table 3:** ANOVA verification of the early warning reaction (first hour) and long-term reaction (10 h) of *Unio tumidus* on digestate application—differences of the mean shell opening level change. Early warning reaction tested between control period (1-h period before digestate application) and mean shell opening level during 10-min intervals after application. Long-term reaction tested between control (1-h period before digestate application) and mean shell opening level during ten 1-h intervals after digestate application

Digestate concentration	Early warning reaction		Reaction during 10-h period	
	F	p	F	p
1:400	308.714	p<0.001	65998.3	p<0.001
1:200	133.685	p<0.001	64591.8	p<0.001
1:100	1507.516	p<0.001	137112.6	p<0.001

**Fig. 2:**
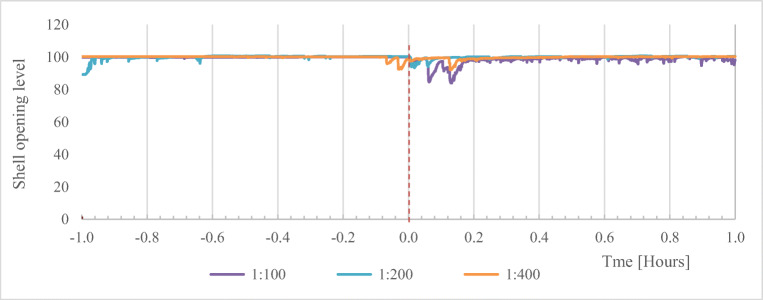
Mussel reaction to digestate application in the concentration 1:400, 1:200, and 1:100 during the first hour. Red line—time of manure application

The general tendency for the mussel reaction to biogas digestate during the first hour is shown graphically in Fig. 2. The curves represent the mean shell opening of eight mussels used in each of the three different biogas digestate dilutions: 1:400, 1:200, and 1:100

During the first 10 min, the shell opening level decreased significantly for all dilutions after applications of biogas digestate compared to the reference value. However, with dilution 1:100 (the highest concentration of biogas digestate), the reaction was stronger (93.9%) compared with the lower concentrations (98.4 and 98.6%). During the following 10-min interval, the shell opening level returned to stand ajar under every digestate concentration. This tendency was also visible during the following time intervals, but mussels remained less open in the largest concentration compared to the pretreatment period (control period). Also, the impact of biogas digestate was very small (less than 1% of the reference value).

### The reaction during the first 10 hours

**Fig. 3:**
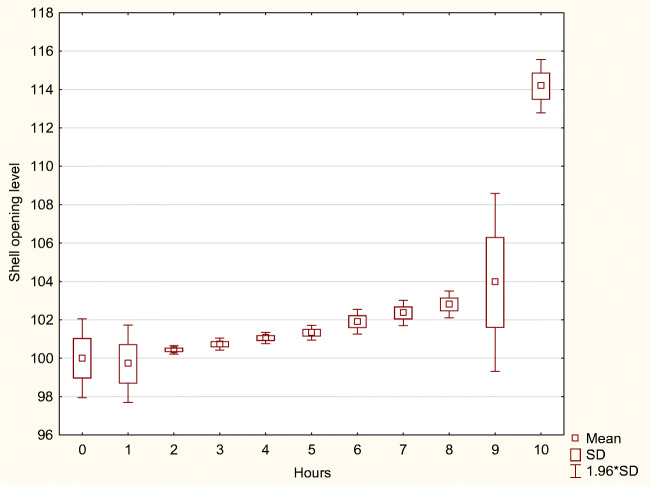
The shell opening level change during the 10-h period after digestate application in the dilutions 1:400

**Fig. 4:**
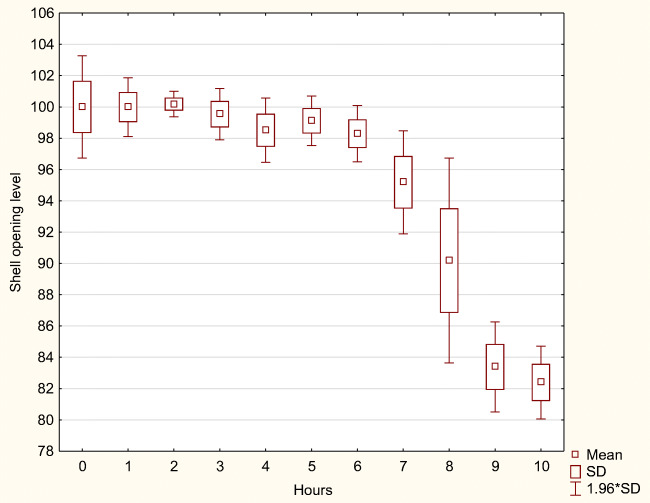
The shell opening level change during the 10-h period after digestate application in the dilutions 1:200

**Fig. 5:**
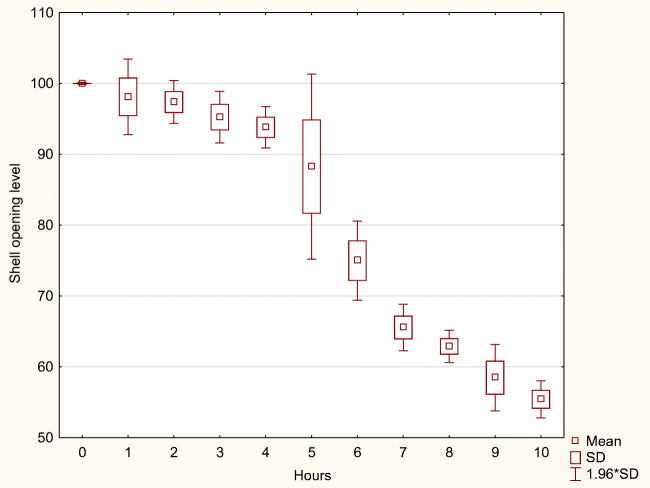
The shell opening level change during the 10-h period after digestate application in the dilutions 1:100

During the 10 h after biogas digestate application, the shell opening changes were analyzed in 1-h intervals. Mean values and variability (standard deviation) are presented in Table 4. Moreso, the tendencies of the mean shell openings are presented graphically (Figs. 3, 4, and 5). ANOVA analysis confirmed that differences between mean values of various time intervals are highly significant (p<0.01) for each digestate concentration (Table 3). The LSD tests undertaken for each difference between means of every 1-h interval appeared significant (p<0.05).

**Table 4:** The shell opening level change during the 10-h period after digestate application. Explanation: *mean*, the mean shell opening level (mean); *SD*, standard deviation; *n*,– number of shell opening measurements recorded; manure dilutions: 1:100, 1:200, and 1:400

Time interval (hours)	n	1:100		1:200		1:400	
		Mean	SD	Mean	SD	Mean	SD
Before application	3189	100.00	0.07	100.00	1.66	100.00	1.05
1st	3191	98.12	2.72	99.99	0.96	99.71	1.03
2nd	3190	97.38	1.54	100.18	0.41	100.44	0.11
3rd	3190	95.24	1.86	99.53	0.84	100.73	0.16
4th	3191	93.81	1.49	98.51	1.05	101.06	0.15
5th	3190	88.27	6.66	99.11	0.81	101.33	0.20
6th	3190	74.99	2.85	98.29	0.91	101.91	0.33
7th	3191	65.55	1.67	95.19	1.68	102.36	0.33
8th	3190	62.90	1.16	90.18	3.34	102.80	0.36
9th	3190	58.47	2.39	83.38	1.47	103.95	2.36
10th	3190	55.42	1.34	82.39	1.19	114.17	0.71
Total	35092	80.92	17.02	95.16	6.60	102.59	3.96

During the 10 h, the shell opening level decreased most strongly for dilution 1:100 (the highest concentration). The decreasing tendency was gradual for every hour and finally reaching 55.4% of the reference value. The strongest drop of the shell ajar was observed during the 5th and 6th hours.

The decreasing tendency was also recorded under the digestate dilution 1:200, but the gradual decreasing tendency was recorded from the 3rd hour after digestate application. The mean shell opening level reached 82.4% of the reference value. The strongest drop of the shell ajar was observed during the 8th hour.

The limited concentration (1:400) did not reduce bivalves' shell openings (except for initial short-term event) but rather, a gradual increasing tendency was observed. The mean shell opening level reached 114.2% of the reference value during the 9th hour after digestate application.

## Discussion

The conducted experimental research allowed us to achieve the aim of the study; to demonstrate changes in the behavioral reaction of the molluscan under the influence of biogas digestate. Studies have shown changes in mussel behavior at different time intervals. Statistical analysis confirmed the relevance of the observations made. Both hypotheses were verified positively by proving that the inflow of biogas digestate has a significant effect on bivalves’ behaviors, and the behavior of bivalves depends on the concentration of biogas digestate content in agricultural wastewater.

Studies have shown that a high concentration of digestate causes a distinct stress response of mussels, resulting in the reduction of shell opening level. It proved that high concentrations cause immediate biological response after biogas digestate application, and the reaction was increasing gradually with time. The decreasing shell opening level proved the stress reaction of bivalves on various kinds of pollution (Bae and Park 2014; Chmist and Szoszkiewicz 2017; Chmist et al. 2019).

This work proved that lower concentrations of biogas digestate cause less stress for mussels—the reaction is delayed and is ultimately weaker. The small dosage of biogas digestate may not cause any stress reactions but indicate a beneficial effect of organic matter delivery. The wide shell opening indicates comfortable conditions, which are probably the result of feeding on digestate organic matter (Newell et al. 2002, 2005). Observing agricultural ecosystems, a positive overall impact of biogas digestate on soil invertebrates as earthworm density and biomass was found (Koblenz et al. 2015), but response of soil biological communities on digestate is still not fully understood (Natalio et al. 2021).

Bivalves’ behavioral response to biogas digestate at high concentration was evident (statistically highly significant) during the entire 10-h experiment. In this way, *Unio tumidus* can deliver bioindication information, which can be useful in water quality monitoring. The behavioral nature of bivalves provides a signal corresponding with the early warning systems based on Hall system technology, which are widely utilized in practice (Hartmann et al. 2016; Sow et al. 2011).

The study focused on detecting biogas digestate, which is becoming a more popular fertilizer. This substance has been used widely in agriculture recently, and its usefulness confirmed in numerous studies (Sogn et al. 2018; Zirkler et al. 2014). The correctly conducted anaerobic digestion delivers biogas digestate of suitable quality for agricultural use (Czekała et al. 2020). Other studies presented threats of agricultural wastes as a significant source of organic substance and nutrient toxins and rich in microorganisms, and pathogenic bacteria (Bień and Nowak 2014). The obtained results have shown that a limited concentration of high-quality digestate does not affect aquatic organisms negatively. Moreover, the study has proven that mussels may be useful in at least preliminary biogas digestate quality assessment. In this way, the complicated and time-consuming chemical and microbiological analysis of biogas digestate is reduced.

The digestate from agricultural biogas plants is used as valuable fertilizer (Czekała et al. 2020). This is due to its richness in macronutrients and micronutrients. The nitrogen content is environmentally particularly important, and therefore, based on this element, the field dose of fertilizer is most often determined. In this way, it is recommended to use a dose not exceeding 170 kg N‧ha^-1^. In practice, 30–40 Mg of digestate is most often used for 1 ha. The analyzed digestate was characterized by the content of 3.73 kg Mg^-1^, and that to maintain below 170 kg N‧ha^-1^, a maximum of about 45 Mg digestate per hectare can be used. However, it should be noted that there may be cases when the nitrogen content is lower, e.g., 2.0, in the research by Głowacka et al. (2020) and higher in Drosg et al. (2015).

Chemical analyses of water during the experiment proved a decrease in nutrient concentration as total forms of phosphorus and nitrogen, and parts of which the bivalves retained as they feed on digestate organic matter (Newell et al. 2002, 2005). Various substances are also accumulated in the gills as a result of water filtration and in the intestine during dietary exposure (Cooper et al. 2010).

The experimental results extended knowledge about bivalves’ behavioral reaction and their suitability in monitoring environmental changes in aquatic environments. Although several scientific studies confirmed the usefulness of various mussel species in water quality monitoring; however, they concerned mostly with toxins (Chmist et al. 2019; Hartmann et al. 2016; Metcalfe and Charlton 1990; Moreira et al. 2019), heavy metals (Salánki et al. 2004), and mineral substances (Chmist and Szoszkiewicz 2017). The biological reaction of mussels to biogas digestate and any kinds of animal manure has not been analyzed so far. The obtained results showed that the behavioral reaction of *Unio tumidus* to digestate pollution is clear and can be used for water quality monitoring. In this way, mussels have the potential to detect manure and other kinds of organic wastes in water.

The importance of this research comes from the tested substance, which is related to biogas production technology, and it plays an increasingly important role when the development of various alternative sources takes place. Since mussels are considered underrepresented in standard ecotoxicological methods (Hartmann et al. 2016) and early warning systems based on the Hall system technology used in these experiments are recognized as promising (Sow et al. 2011). We anticipate that our results can have important contributions to the development of a monitoring system, controlling the risk of water contamination during biogas production.

## Conclusions

The inflow of biogas digestate might cause a significant stress reaction of freshwater bivalves. The behavioral reaction of bivalves depends on the concentration of biogas digestate in wastewater—a rise in concentration reduces shell opening level. The behavioral reaction of *Unio tumidus* might be utilized in the early warning systems to detect threats of organic waste pollution in water. Freshwater bivalves maybe use as a primary method for assessing the risk of water contamination during biogas production.

## Data Availability

The data that support the findings of this study are available from the corresponding author upon reasonable request in the forms of raw data, samples, and records.

## References

[CR1] Bae MJ, Park YS (2014). Biological early warning system based on the responses of aquatic organisms to disturbances: a review. Sci Total Environ.

[CR2] Bień J, Nowak D (2014). Biological composition of sewage sludge in the aspect of threats to the natural environment. Arch Environ Pro.

[CR3] Błachuta J, Szoszkiewicz K, Gebler D, Schneider SC (2014). How do environmental parameters relate to macroinvertebrate metrics – prospects for river water quality assessment. Pol J Ecol.

[CR4] Brabec K, Szoszkiewicz K (2006). Macrophytes and diatoms – major results and conclusions from the STAR project. Hydrobiologia.

[CR5] Chmist J, Szoszkiewicz K (2017). Attempt at assessment of *Unio tumidus* bivalve mollusks suitability for monitoring water iron content. Ochr Srodowiska.

[CR6] Chmist J, Hamerling M, Szoszkiewicz K (2018). Choice of the most useful biological early warning system based on AHP and Rembrandt analysis. Acta Sci Pol.

[CR7] Chmist J, Szoszkiewicz K, Drożdżyński D (2019). Behavioural responses of *Unio tumidus* fresh water mussel to pesticide contamination. Arch Environ Contam Toxicol.

[CR8] Cooper S, Hare L, Campbell PG (2010). Subcellular partitioning of cadmium in the freshwater bivalve, Pyganodon grandis, after separate short-term exposures to waterborne or diet-borne metal. Aquat Toxicol.

[CR9] Czekała W (2019). Biogas Production from Raw Digestate and its Fraction. Ecol Eng.

[CR10] Czekała W, Dach J, Dong R, Janczak D, Malińska K, Jóźwiakowski K, Smurzyńska A, Cieślik M (2017). Composting potential of the solid fraction of digested pulp produced by a biogas plant. Biosyst Eng.

[CR11] Czekała W, Lewicki A, Pochwatka P, Czekała A, Wojcieszak D, Jóźwiakowski K, Waliszewska H (2020). Digestate management in polish farms as an element of the nutrient cycle. J Clean Prod.

[CR12] Drosg B, Fuchs W, Al Seadi T, Madsen M, Linke B. (2015) Nutrient Recovery by Biogas Digestate Processing. EA Bioenergy. http://task37.ieabioenergy.com/files/daten-redaktion/download/Technical%20Brochures/NUTRIENT_RECOVERY_RZ_web1.pdf

[CR13] Głowacka A, Szostak B, Klebaniuk R (2020). Effect of biogas digestate and mineral fertilisation on the soil properties and yield and nutritional value of switchgrass forage. Agronomy.

[CR14] Hartmann JT, Beggel S, Auerswald K, Stoeckle BC, Geist J (2016). Establishing mussel behavior as a biomarker in ecotoxicology. Aquat Toxicol.

[CR15] Jakubik B, Lewandowski K (2016). Co można odczytać z muszli mięczaka?. Kosmos.

[CR16] Koblenz S, Tischer S, Rücknagel J, Christen O (2015). Influence of biogas digestate on density, biomass and community composition of earthworms. Ind Crop Prod.

[CR17] Kramer KJ, Foekema EM, Butterworth FM, Gunatilaka A, Gonsebatt ME (2001). The “Musselmonitor” as Biological Early Warning System. Biomonitors and biomarkers as indicators of environmental change.

[CR18] Kramer KJM, Foekema EM, Butterworth FM, Gunatilaka A, Gonsebatt ME (2001). The “Musselmonitor®” as Biological Early Warning System. Biomonitors and biomarkers as indicators of environmental change 2. Environmental Science Research.

[CR19] Li L, Binghui Z, Lusan L (2010). Biomonitoring and bioindicators used for river ecosystems: definitions, approaches and trends. Procedia Environ Sci.

[CR20] Mazur R, Shubiao W, Szoszkiewicz K, Bedla D, Nowak A (2016). A *Lymnaea stagnalis* embryo test for toxicity bioindication of acidification and ammonia pollution in water. Water.

[CR21] Mazur R, Szoszkiewicz K, Lewicki P, Bedla D (2018). The use of computer image analysis in a *Lemna minor* L. bioassay. Hydrobiologia.

[CR22] Metcalfe JL, Charlton MN (1990). Freshwater mussels as biomonitors for organic industrial contaminants and pesticides in the St. Lawrence River. Sci Total Environ.

[CR23] Moreira BL, Sasaki TS, Taniguchi S, Bícego CM, Leticia VC, Abessa DM (2019). Impacts of dredging on biomarkers responses of caged bivalves in a semi-arid region (Ceará State, NE Brazil). Mar Environ Res.

[CR24] Nag R, Whyte P, Markey BK, O'Flaherty V, Bolton D, Fenton O, Richards KG, Cummins E (2020). Ranking hazards pertaining to human health concerns from land application of anaerobic digestate. Sci Total Environ.

[CR25] Natalio AIM, Back M, Richards A, Jeffery S (2021). The effects of saline toxicity and food-based AD digestate on the earthworm Allolobophora chlorotica. Geoderma.

[CR26] Newell RI, Cornwell JC, Owens MS (2002). Influence of simulated bivalve biodeposition and microphytobenthos on sediment nitrogen dynamics. Limnol Oceanogr.

[CR27] Newell RI, Fisher TR, Holyoke RR, Cornwell JC (2005). Influence of eastern oysters on nitrogen and phosphorus regeneration in Chesapeake Bay, USA. The comparative roles of suspension-feeders in ecosystems.

[CR28] Parmar TK, Rawtani D, Agrawal YK (2016). Bioindicators: the natural indicator of environmental pollution. Frontiers in Life Science.

[CR29] Regulation of Ministry of Agriculture and Rural Development of 18 June 2008 on the implementation of certain provisions of the act on fertilizers and fertilization (2008) (Dz.U. nr 119 poz. 765) (in Polish).

[CR30] Salánki J, Farkas A, Kamardina T, Rózsa KS (2003). Molluscs in biological monitoring of water quality. Toxicol Lett.

[CR31] Schöne BR, Krause RA (2016). Retrospective environmental biomonitoring–mussel watch expanded. Glob Planet Chang.

[CR32] Sogn TA, Dragicevic I, Linjordet R, Krogstad T, Eijsink GH, Eich-Greatorex S (2018). Recycling of biogas digestates in plant production: NPK fertilizer value and risk of leaching. Int J Recycl Org Waste Agricult.

[CR33] Sow M, Durrieu G, Briollais L, Ciret P, Massabuau JC (2011). Water quality assessment by means of HFNI valvometry and high-frequency data modeling. Environ Monit Assess.

[CR34] Vinas L, Besada V, Sericano J (2012). 1.19 - Sampling of fish, benthic species, and seabird eggs in pollution assessment. Comprehensive Sampling and Sample Preparation.

[CR35] Zhou Q, Zhang J, Fu J, Shi J, Jiang G (2008). Biomonitoring: an appealing tool for assessment of metal pollution in the aquatic ecosystem. Anal Chim Acta.

[CR36] Zirkler D, Peters A, Kaupenjohann M (2014). Elemental composition of biogas residues: Variability and alteration during anaerobic digestion. Biomass Bioenergy.

